# Electroclinical Features of Epilepsy in Mucopolysaccharidosis III: Outcome Description in a Cohort of 15 Italian Patients

**DOI:** 10.3389/fneur.2021.705423

**Published:** 2021-07-19

**Authors:** Rita Barone, Agata Fiumara, Mariangela Gulisano, Lara Cirnigliaro, Maria Donatella Cocuzza, Claudia Guida, Fabio Pettinato, Filippo Greco, Maurizio Elia, Renata Rizzo

**Affiliations:** ^1^Child Neurology and Psychiatry Section, Department of Clinical and Experimental Medicine, University of Catania, Catania, Italy; ^2^Regional Referral Centre for Inborn Errors Metabolism, University Children Hospital, Policlinico San Marco, Catania, Italy; ^3^Paediatric Section, Department of Clinical and Experimental Medicine, University of Catania, Catania, Italy; ^4^Oasi Research Institute, Istituto di Ricerca a Carattere Scientifico (IRCCS), Troina, Italy

**Keywords:** mucopolysaccharidosis type III, sanfilippo syndrome, epilepsy, electroencephalogram, outcome

## Abstract

Mucopolysaccharidosis III (Sanfilippo syndromes) types A–D are rare lysosomal storage disorders characterized by heparan sulfate accumulation and neurodegeneration. Patients with MPS III present with developmental stagnation and/or regression, sleep disturbance, and behavioral abnormalities usually in the first years of life. Epilepsy may occur in a proportion of patients during the disease course. However, the progression of epilepsy and EEG changes in MPS III have not been systematically investigated. We report electroclinical features in a cohort of patients with MPS III over a follow-up period ranging from 6.5 to 22 years. Participants include 15 patients (11 females; aged 7–31 years) with MPS III A (*n* = 7, 47%), MPS III B (*n* = 5, 34%), MPS III C (*n* = 2, 13%), and MPS III D (*n* = 1, 6%). At the time of this study, 8 out of 15 patients (53%) had epilepsy. Epilepsy occurred in patients with advanced disease even in the first decade of life (mean age at onset: 12.1 ± 6.7 years). However, seizure onset may also be associated with abrupt worsening of the neurobehavioral phenotype. The main epilepsy types observed were generalized (four out of eight, 50%), followed by focal (three out of eight, 37%) and combined (two out of eight, 25%) epilepsy and status epilepticus (one out of eight, 12.5%). Seizures were generally controlled by one antiepileptic drug (AED) and most patients (seven out of eight, 87%) were still on therapy after a median follow-up period of 5 years (range: 1–9 years). A total of 66 EEGs were analyzed with a median EEG follow-up duration of 7 years (range: 6 months−14 years). Slowing of the background activity occurred in 7 (46%) patients aged 4–19 years. Epileptiform EEG abnormalities were observed in 10 patients at a mean age of 9.6 ± 2.9 years. EEG epileptiform discharges were not unavoidably linked to epilepsy. Early recognition and careful monitoring of electroclinical features in MPS III is necessary for appropriate care and for the detection of disease progression.

## Introduction

Lysosomal storage disorders (LSD) are inborn errors of metabolism caused by a deficiency of lysosomal function. In LSD, the undegraded storage material triggers complex pathogenic cascades that are responsible for disease pathology (1). Mucopolysaccharidoses (MPS) are a group of rare inherited metabolic disorders caused by deficiency of specific glycosaminoglycan (GAG)-degrading lysosomal enzymes, leading to intracellular GAG accumulation and progressive multi-organ dysfunction. The central nervous system is primarily affected in neuronopathic forms as MPS I (Hurler syndrome), MPS II, MPS III, and MPS VII (2). Mucopolysaccharidosis type III (MPS III, Sanfilippo syndrome) includes four autosomal recessive lysosomal storage disorders (MPS III A–D), caused by a deficiency in one of the four enzymes involved in the degradation of the glycosaminoglycan heparan sulfate. Based on a recent systematic review, the overall prevalence is 0.17–2.35 per 100,000 live births for all four subtypes of MPS III combined (3). Among all subtypes, types A and B are more frequent than types C and D. The progressive storage of heparan sulfate in the MPS III brain is associated with neuroinflammation and oxidative stress, protein misfolding, cellular signaling defects, and impairment of autophagy causing loss of neurons as well as cognitive and motor decline (4). MPS III is characterized by developmental delay, impaired cognition, behavioral problems, and sleep disturbances (5, 6) and by the possible onset of epileptic seizures (7). Three stages were proposed to describe the clinical evolution of classical MPS III: the first phase usually starts between 1 and 2 years with developmental delay, in particular with speech delay; the second phase begins around 2–4 years as patients present with behavioral problems, sleep disturbance, and progressive intellectual decline; and the third phase usually begins in the teenage years with progressive decline of motor abilities and loss of ambulation (8, 9).

The MPS III clinical phenotype is heterogeneous with variable age of onset (median age of 2.5 years) and diverse rate of progression (10–14). The great phenotypic variability appears to reflect, in part, the large allelic heterogeneity with resulting differences in the residual enzymatic activity (10, 11). Patients usually die at the end of the second decade, although longer survival has been reported in patients with MPS III C and III D and in the attenuated phenotypes of MPS III A (15) and III B (15, 16). Currently, no effective therapy is available to slow down or reverse neurodegeneration in MPS III. Different therapeutic strategies have been tested during recent years in cellular and animal models of the disease and in clinical trials (17). Symptomatic therapy is thus the mainstay of treatment to ameliorate the quality of life of patients and their relatives.

Despite the high prevalence (26–52%) and debilitating nature of epilepsy in MPS III patients (7), current knowledge on the clinical and electroencephalographic (EEG) features of MPS III-related epilepsy over the disease course remains limited.

In order to systematically investigate the electroclinical outcome of epilepsy in MPS III, we analyzed the electroclinical features of patients with MPS III diagnosed over a 24-year period. As seizures, epileptiform EEG activity, and changes in cognition and behavior seem to be intertwined, we searched for possible mutual interaction between these manifestations.

## Methods

This retrospective study was based solely on information and investigations that were carried out as part of the routine clinical care of patients with MPS III. Access to the clinical files for data extraction was obtained after written informed consent was signed by the parents. We included patients with an MPS III (Sanfilippo syndrome) diagnosis seen from January 1996 to December 2020 at the Referral Center for inherited metabolic diseases, University Hospital of Catania (Italy). The diagnosis of MPS III was confirmed by urinary GAG measurements and specific enzyme assays performed in skin fibroblasts. Genetic findings were included when available. Inclusion criteria were as follows: proven MPS III diagnosis, availability of detailed clinical history with particular regard to epilepsy, and availability of at least one EEG recording during the disease course. Collection of clinical data included demographic data, age at seizure onset if any, seizure semiology, neurobehavioral phenotype at the time of seizure onset, and epilepsy treatment and outcome. Routine EEG (interictal and ictal) and sleep-deprived ambulatory EEG were performed at the Child Neuropsychiatry ward, University Hospital Catania and/or at the IRCCS “Oasi” Troina (Italy). EEGs were obtained at different points during the disease course depending on the presence of seizures and/or availability of patients. EEG recordings were independently analyzed by two independent pediatric neurologists (ME and MDC) with long-standing expertise in childhood epilepsy. Response to antiepileptic drug (AED) therapy was based on clinical evaluation and reports of parents.

## Results

### Clinical Characteristics

Participants include 15 patients (11 females; aged 7–31 years) from 12 unrelated Sicilian families, with proven MPS III diagnosis: MPS III A (N-sulfoglucosamine sulfohydrolase deficiency) (*n* = 7, 47%), III B (N-acetyl-glucosaminidase deficiency) (*n* = 5, 34%), MPS III C (*n* = 2, 13%) (heparan-alpha-glucosaminide N-acetyltransferase deficiency), and MPS III D (*n* = 1, 6%) (N-acetylglucosamine-6-sulfatase deficiency) (Table 1). Patients comprised three pairs of sibs, with MPS III A, III B, and III C, respectively. Parental consanguinity was ascertained in two families. Presenting symptoms included developmental delay in particular with speech delay, sleep disturbance and behavioral problems, mild facial coarsening, and variable occurrence of hepatomegaly and/or skeletal problems. Age at confirmed diagnosis for all 15 patients was 3.4 ± 2.3 years (mean ± SD) (median: 3; range: 6 months to 9 years). The youngest age at diagnosis (6 months) was in the younger affected sib with MPS III B (ID# 8). The oldest age at diagnosis was in two patients with MPS III C (9 years) and III D (8 years), respectively. The mean age at last visit was 15.6 ± 5.7 (median: 14; range: 7–31 years) with a median follow-up duration of 11 years (range: 6.5–22 years). At the time of the study, five patients (three with MPS III A and two with MPS III B) had passed away at a mean age of 17.4 ± 3.2 years (median: 17; range: 14–22) due to cardiorespiratory failure.

**Table 1:** Demographic, molecular, and neurobehavioral features in patients with MPS III.

**ID**	**MPS III**	**Sex**	**Age at diagnosis**	**Genetic findings**	**Age at last visit/deceased (y)**	**Epilepsy/ onset (y)**	**Seizure type**	**AED tried**	**Neurobehavioral features at seizure onset^a^**	**Brain MRI findings at seizure onset^a^**
1	A	M	24 m	c.1079delC (p.V361fsX52)/ c.1079delC (p.V361fsX52)	13/+ (17)	+/12	GTCS	LAC	Unsteady gait, severe ID, absent speech, temper tantrums	Global cerebral and cerebellar atrophy
2	A	F	12 m	c.1079delC (p.V361fsX52)/ c.1079delC (p.V361fsX52)	12/–	+/8	GTCS	VPA, LEV	Unsteady gait, severe ID, absent speech, temper tantrums, motor stereotypes	Global cerebral and cerebellar atrophy
3	A	F	36 m	c.220C>T (p.R74C)/ c.364>A (p.G122R)	14/–	+/7	FMSWA	CBZ	Unsteady gait, severe ID, absent speech, temper tantrums, autistic features	Global cerebral and cerebellar atrophy, white matter changes, corpus callosum atrophy
4	A	F	20 m	n.a.	12/+ (15)	+/12	GTCS	VPA	Walk with support, lower limb spasticity. Severe ID, absent speech	Diffuse cortical atrophy and corpus callosum atrophy
5	A	F	48 m	c.197C>G (p.S66W)/ c.220C>T (p.R74C)	18/–	+/11	FSWA	CBZ	Unsteady gait. Brisk tendinous reflexes. Severe ID, absent speech, self-injurious behavior	Global cerebral and cerebellar atrophy
6	A	F	30 m	n.a.	7/+ (9)	–	–	–	Unable to stand. Severe ID, absent speech, autistic features^a^	Global cerebral and cerebellar atrophy n.a.
7	A	F	40 m	n.a.	18/–	–	–	–	Unable to walk unaided, severe ID, absent speech, autistic features^a^	Global cerebral and cerebellar atrophy
8	B	F	6 m	c.874G>A (p.G292R)/ c.1928G>A (p.R643H)	12/–	–	–	–	Independent walking. Severe ID, word-sentence speech, hyperactivity, temper tantrums	Global cerebral and cerebellar atrophy, white matter changes
9	B	F	36 m	c.874G>A (p.G292R)/ c.1928G>A (p.R643H)	14/–	–	–	–	Unsteady gait. Severe ID Absent speech, temper tantrums	Global cerebral and cerebellar atrophy, white matter changes
10	B	M	18 m	c.874G>A (p.G292R)/ c.874G>A (p.G292R)	18/+ (19)	+/9	AS-GTCS-SE	VPA, TPM PB, CLN, LEV	Unable to walk. Upper and lower limb spasticity. Severe ID Absent speech	Cerebral and cerebellar atrophy
11	B	M	36 m	c.274T>C (p.Y92H)/ c.274T>C (p.Y92H)	19/–	–	–	CLN	Unable to sit. Upper and lower limb spasticity. Cervical dystonia, tremors Severe ID, absent speech	Cerebral and cerebellar atrophy
12	B	F	50 m	n.a.	15/+ (22)	+/9	GTCS	VPA	Unsteady gait, severe ID, absent speech, autistic features	Cerebral and cerebellar atrophy, white matter changes
13	C	F	9y	c.852-1G>A/ c.852-1G>A	31/–	+/28	FMSWA GTCS	LAC	Unable to walk. Lower limb spasticity. Severe ID, absent speech, motor stereotypes	Cerebral and cerebellar atrophy, white matter changes
14	C	M	6y	c.852-1G>A/ c.852-1G>A	29/–	–	–	–	Unsteady gait. Severe ID, absent speech	Cerebral and cerebellar atrophy, white matter changes
15	D	F	8y	c.1019>G (p.K340R)/ c.1019>G (p.K340R)	9/–	–	–	–	Able to walk, moderate ID, impaired speech	–

*Sib pairs: patients ID# 1–2, 8–9, and 13–14*.

*M, male; F, female; m, months; y, years; –, absent; +, present; n.a., not available; MRI, magnetic resonance imaging; AS, absence seizures; FSWA, focal seizure with impairment of awareness; FMSWA, focal motor seizure with impairment of awareness; GTCS, generalized tonic–clonic seizure; SE, status epilepticus; CBZ, carbamazepine; CLN, clonazepam; LAC, lacosamide; LEV, levetiracetam; PB, phenobarbital; TPM, topiramate; VPA, valproic acid*.

a *In patients with no epilepsy, reported neurobehavioral features and MRI findings refer to the last examination*.

### Epilepsy Clinical Features

**Figure 1:**
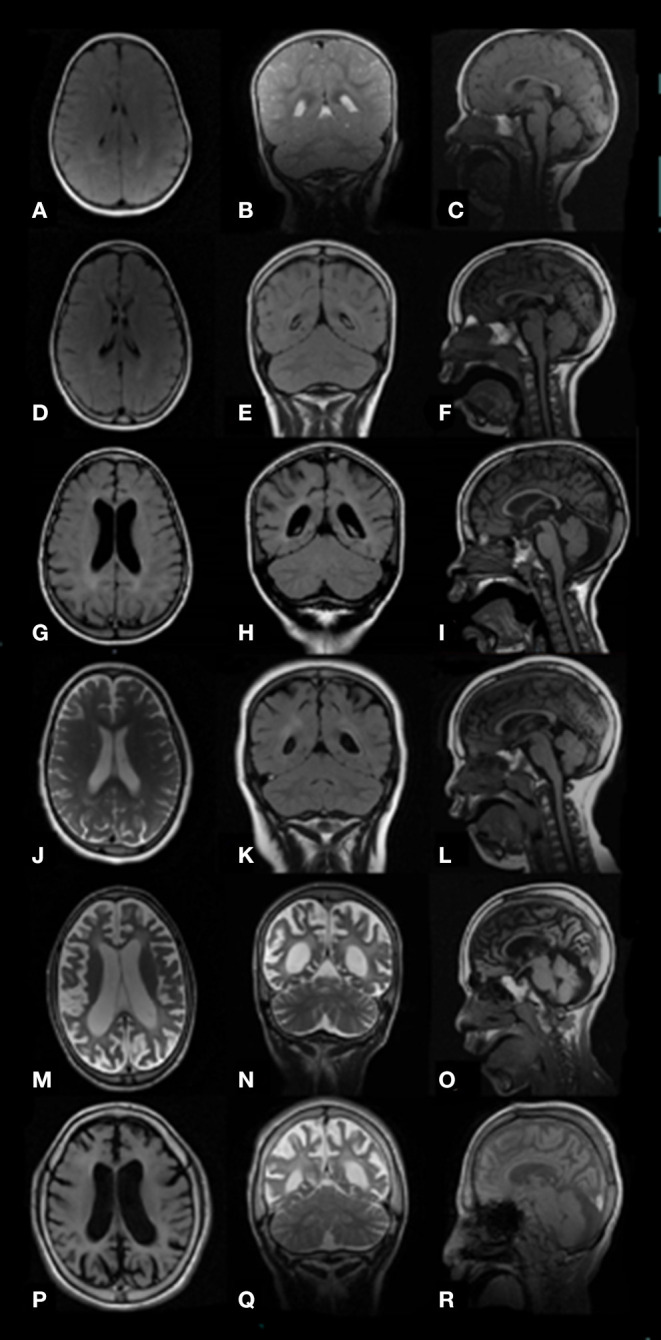
Brain images MRI (axial T2 or FLAIR, coronal T2 or FLAIR, and sagittal T1) of study patients with MPS III at different ages show progressive atrophic changes with enlargement of the lateral ventricles and progressive widening of the sulci secondary to brain volume loss. Pts. ID# 8 MPS III B, ages 19 months **(A–C)** and 6 years **(D–F)**; ID# 3 MPS III A, age 7 years **(G–I)**; ID# 9 MPS III B, age 8 years **(J–L)**; ID# 3 MPS III A, age 11 years **(M–O)**; ID# 10 MPS III B, age 12 years **(P–R)**.

At study initiation, 8 out of 15 patients (53%) had epilepsy (Table 1). The mean age at first seizure was 12.1 ± 6.7 years (median: 10; range: 7–28 years). Four subjects developed seizures before the age of 10 years, three between 10 and 20 years of age, and one patient with MPS III C in adulthood. Febrile (simple or complex) seizures were not reported. The main epilepsy type was generalized epilepsy (four out of eight, 50%), followed by focal (three out of eight, 37%) and combined (two out of eight, 25%) epilepsy and status epilepticus (one out of eight, 12.5%). Six subjects (ID# 1, 2, 4, 10, 12, 13) (75%) had generalized tonic–clonic seizures (GTCS) and three subjects (ID# 3, 5, 13) (37%) had focal motor or nonmotor seizures with impairment of awareness. Seizure frequency ranged from daily (ID# 2, 10, 12, 13) to occasional (ID# 1, 3, 4, 5). One patient (ID# 10) experienced repeated episodes of status epilepticus by his late teens. All patients with epilepsy underwent therapy with AEDs (Table 1). Valproic acid, carbamazepine, levetiracetam, lacosamide, and benzodiazepines, alone or in combination, were the most commonly used and effective drugs. Seizures were controlled by one AED in almost all patients except for the MPS III B (ID# 10) patient with drug-resistant daily GTCS and recurrent non-convulsive status epilepticus (SE). Out of eight patients with epilepsy, seven (87%) were still on therapy after a median follow-up period of 5 years (range: 1–9 years). Among these patients, most (five out of seven, 71%) were seizure-free, whereas two patients (ID# 10 and 13) still suffered from recurrent seizures. One patient (ID# 5) with focal seizures remained seizure-free after therapy discontinuation over a 7-year period. Brain magnetic resonance imaging (MRI) was repeated along the disease course and showed progressive cerebral and cerebellar atrophy, corpus callosum atrophy, and variable periventricular white matter changes (Figure 1).

### EEG Findings

Primary EEG characteristics are reported in Tables 2, 3. In all, 66 EEGs (82% during wakefulness) were analyzed (Table 2). All patients had at least one EEG recording. EEG during sleep was recorded at least once in seven patients. In 11 patients, EEGs were obtained at different points during the disease course, depending on the presence of seizures and the compliance of patients. The median EEG follow-up duration was 7 years (range: 6 months−14 years). In seven (46%) patients aged from 4 to 19 years, awake EEG showed slowing of the background activity in the theta–delta range with deficit of the physiological anteroposterior slow–fast gradient. During sleep EEG recording, poor or no differentiation in sleep–wake cycle was observed in five out of seven studied patients at ages ranging from 4 to 15 years. Epileptiform EEG abnormalities were observed in 10 patients at a mean age of 9.6 ± 2.9 years (median: 9.5; range: 5–12 years) (Table 2). Generalized sharp waves and spike-and-wave complexes were recorded in five patients (50%) (ID# 3, 5, 8, 12, 14). Multifocal and generalized sharp waves and spike-and-wave complexes were observed in three patients (30%) (ID# 2, 10, 13). Bilateral multifocal asynchronous spikes were evident in two patients (20%) (ID# 4, 6). Out of 10 patients with EEG epileptic activity, eight had epilepsy (Table 3). Epileptiform EEG activity was not associated with seizures in one patient with MPS III B (ID# 8) at age 5 as well as in a patient with MPS III C (ID# 14) at 12 years of age. Moreover, one individual with MPS III A (ID# 2) on treatment with valproate since 8 years of age was seizure-free and without epileptiform discharges in the last EEG recording at age 12 years.

**Table 2:** Overall EEG findings in patients with mucopolysaccharidosis type III (*N* = 15).

**Number of recorded EEGs**
*N* (range)	66	(1–11)
Mean ± SD	4.4	3.4
EEG type	*N*	(%)
Wakefulness	55	82
Wakefulness and sleep	11	16
**Background activity**
Slow	7	46
Normal	8	53
Epileptiform abnormalities^*^	10	66
Focal	1	6
Multifocal	5	33
Generalized	8	53

*EEG, electroencephalogram*.

* *More than one type of abnormalities could be present in the same subject*.

**Table 3:** EEG features over the disease course in patients with MPS III.

**Patient ID**	**MPS type**	**Age at first EEG (y)**	**Age at last EEG (y)**	**EEG follow-up duration (y)**	**Studied EEG (N)**	**EEG epileptiform activity/age (y)**	**Epileptiform interictal activity**	**Background activity**	**Epilepsy/age at onset (y)**	**Paroxysmal activity at last EEG follow-up/age**
1	III A	12	12.5	0.5	2	+/12	Multifocal spikes and generalized spike-and-wave and polyspike-wave complexes	Normal	+/12	–/12.5^*^
2	III A	12	12	–	1	–	–	Normal	+/8	–/12^*^
3	III A	7	14	7	4	+/7	Generalized sharp waves and spike-and-wave complexes	Slowing	+/7	+/14^*^
4	III A	10	13	3	4	+/10	Multifocal spike-and-waves	Normal	+/12	–/11^*^
5	III A	4	18	14	8	+/9	Generalized sharp waves	Normal	+/11	–/14
6	III A	4	9	5	4	+/7	Multifocal spike-and-waves	Slowing	–	–/9
7	III A	18	18	–	1	–	–	Normal	–	–/18
8	III B	1	9	8	5	+/5	Generalized spike-and-wave and polyspike-wave complexes	Normal	–	–/7
9	III B	4	11	7	3	–	–	Slowing	–	–/11
10	III B	10	18	8	7	+/10	Multifocal and generalized sharp waves and spike-and-wave complexes–recurrent generalized and frontal focal spikes	Slowing	+/9	+/18^*^
11	III B	19	19	–	1	–/19	–	Slowing	–	–/19
12	III B	9	15	6	3	+/9	Generalized sharp waves and spike-and-wave complexes	Slowing	+/9	+/15^*^
13	III C	15	31	12	11	+/15	Multifocal and generalized spikes and spike-and-wave complexes	Normal	+/28	+/31^*^
14	III C	12	24	12	11	+/12	Generalized sharp waves and spike-and-wave complexes	Normal	–	+/24
15	III D	9	9	–	1	–/10	–	Slowing	–	–/9

* *Patients (ID# 1, 2, 3, 4, 10, 12, 13) were on treatment with AED at last EEG follow-up (see Table 1 for tried AED)*.

*+, present; –, absent; y, years; EEG, electroencephalogram*.

## Discussion

In the present study, we evaluated the electroclinical features of epilepsy in 15 patients with MPS III over a disease period ranging from 6.5 to 22 years. Epilepsy occurred in almost half of the study patients (53%) at a median age of 10. All patients were 7 years or older at epilepsy onset, when deterioration of neurocognitive function was already advanced. Four subjects developed seizures before age 10, including three patients with MPS III A and one with MPS III B. Previous studies showed that on average, epilepsy is present in almost half of patients with MPS III (7, 12, 18). Most MPS III A and III B patients manifest epilepsy at the end of their first decade, whereas for MPS III C, this might occur also at a later age (10, 19). Moreover, patients with a severe MPS III phenotype developed epilepsy at a significantly earlier age, compared with patients suffering from attenuated MPS III A or MPS III B (15, 16). In a Dutch study, out of 20 patients with attenuated MPS III B (age 18–63 years), 10 patients developed seizures at a median age of 34.1 years. Most of these patients had GTCS and half of them were seizure-free under treatment with anti-epileptic drugs (20). Although MPS III C is thought to have a milder course than other MPS III types, epilepsy was reported in up to half of the studied patients (45%) (10). However, epilepsy occurred at a later age in a series of patients with MPS III C, usually in adulthood, with a mean age at onset of 23 years (19), which is consistent also with the present study (patient ID# 13). Convulsions in MPS III are mainly GTCS (8, 12, 20). Combined seizure type with focal seizures may occur (21) as well as unusual seizure patterns, such as nocturnal bouts of laughing or “panting” (8). According to the new ILAE classification of seizures and epilepsy (22), in our series, the main epilepsy type was generalized epilepsy (75%) followed by focal epilepsy (37%). In particular, most patients suffered from GTCS, while three subjects exhibited focal motor or non-motor seizures with impairment of awareness. In one girl with MPS III A (ID# 5), focal seizures appeared at age 11 with recurrent episodes of conjugate deviation of the eyes to the left and impairment of awareness. Seizure onset was associated with acute deterioration of her behavioral pattern with restlessness and self-injurious behavior and sleep disturbances (such as frequent nocturnal wakening) followed by repetitive, stereotyped, and rhythmic movements (leg banging). In this patient, low-dose carbamazepine therapy (200 mg per day) was followed by rapid behavioral amelioration (decrease of hyperactivity), reduction of seizure frequency, and nocturnal wakening. Bonanni et al. described a patient with MPS III A that exhibited profound cognitive impairment, impaired speech, hearing loss, uncontrollable hyperactivity, and sleep disturbances at the age of 11. The sleep was disturbed by recurring episodes of sudden wakening and chaotic motor activity and by frequent stereotyped attacks of motor automatism (pedaling), rotation of the trunk, vocalization, and frightened expression consistent with a diagnosis of nocturnal frontal lobe epilepsy (21). Based on the present and the abovementioned study (21), differentiation of abnormal paroxysmal motor events in sleep and parasomnias is challenging in patients with a neurodegenerative disorder, such as MPS III and epilepsy treatment may play a role in controlling associated behavioral and sleep disturbances in these patients. We found that epilepsy was controlled by AED monotherapy in the majority of patients. However, almost all (seven out of eight) patients were still on therapy after a median follow-up period of 5 years (range: 1–9 years). One patient with MPS III B (ID# 10) had drug-resistant daily generalized seizures and recurrent non-convulsive SE. In this patient, refractory epilepsy onset with absence seizures at age 9 was associated with loss of speech and walking ability. Recurrent, non-convulsive SE manifested for the first time at age 10 with lack of responding to his name and with more severe disconnection from his environment. Ictal EEG recording showed generalized, subcontinuous spike waves followed by a sustained frontal epileptic activity that subsided with phenobarbital and clonazepam add-on therapy. Non-convulsive SE previously described in MPS II (21) and in MPS III (18) may complicate the course of patients with neuronopathic MPS and may be difficult to recognize owing to severe neurobehavioral impairment in these patients.

In the present study, EEG showed moderate slowing of background activity in seven (46%) patients and scarce differentiation of the sleep–wake cycle in five out of seven studied patients (71%), with the youngest patients being 4 years old at the time of study. It appears that EEG changes without epileptiform activity may convey information on the early phase of the neurodegenerative course in patients with MPS III. In this regard, Husain et al. found that among 13 patients with MPS III at a mean age of 2.7 years, EEG showed diffuse slowing in 6 (46%), thus suggesting that diffuse slowing without epileptiform abnormalities may represent the most common EEG feature in the early phase of MPS III (23). We found epileptiform EEG abnormalities in 10 patients (66%) at median age of 9.5 (range: 5–12 years). According to previous studies, generalized epileptiform activity was most frequently observed, followed by multifocal and combined epileptic discharges (7, 13, 21). Interestingly, epileptiform EEG activity was not associated with seizures in one patient with MPS III B (ID# 8) at age 5 as well as in a patient with MPS III C (ID# 14) at 12 years of age. Thus, we highlight that epileptogenic paroxysms may be frequently found in children with MPS III with or without epilepsy. The present study shows some limitations and it should be viewed in the context of the following considerations: first, the relatively low number of study patients was entirely derived from a single academic center and is not intended to be representative of all patients with MPS III. In particular, we did not examine MPS III patients with attenuated phenotypes which have a milder disease course. Second, our findings were retrospectively collected. Thus, systematic investigations on newly diagnosed patients might be helpful to define clinical predictors of epilepsy and EEG changes prospectively.

## Conclusions

In this study, we examined the course of epilepsy and EEG findings in a series of patients with MPS III diagnosed over a 24-year period. Epilepsy typically presented with the features of GTCS. Absence epilepsy, focal seizure with impaired awareness, and non-convulsive SE were also observed. Epilepsy occurred in patients with advanced disease even in the first decade of life, and seizure onset might be associated with abrupt worsening of the neurobehavioral phenotype. We underline that detection of epileptic activity, including non-convulsive status epilepticus in MPS III patients, can be difficult as subtle seizures may be missed in patients with cognitive deficits and behavioral problems. EEG changes, specifically slowing of the background rhythm and poor differentiation of the sleep–wake cycle without epileptiform EEG activity, may convey information on the early phase of the neurodegenerative course in patients with MPS III. Epileptiform EEG abnormalities were frequently observed and they were not unavoidably linked to epilepsy. AEDs were effective in controlling seizures in patients with MPS III and most treated patients were seizure-free in long-term follow-up. Based on these data, early recognition and careful monitoring of the electroclinical features in MPS III may be necessary for appropriate care and for the detection of disease progression.

## Data Availability Statement

The original contributions presented in the study are included in the article, further inquiries can be directed to the corresponding author/s.

## Ethics Statement

Ethical review and approval was not required for the study on human participants in accordance with the local legislation and institutional requirements. Written informed consent to participate in this study was provided by the participants' legal guardian/next of kin.

## Author Contributions

RB conceptualized the work and wrote the original draft. AF and RB performed the diagnosis of MPS in the study patients. AF, RB, FG, and FP performed the clinical follow-up. RB, LC, MG, and FP collected and analyzed the data. MDC, ME, and CG performed and evaluated EEG recordings. ME analyzed the EEG data. RB and LC revised the pertinent literature. AF and RR were involved in planning and supervised the work. RB, AF, ME, and RR revised and edited the manuscript. All authors have agreed to this final version and participated in a meaningful way in the preparation of the manuscript.

## Conflict of Interest

The authors declare that the research was conducted in the absence of any commercial or financial relationships that could be construed as a potential conflict of interest.
